# Changes in the Strength Properties and Phase Transition of Gypsum Modified with Microspheres, Aerogel and HEMC Polymer

**DOI:** 10.3390/ma14133486

**Published:** 2021-06-23

**Authors:** Justyna Ciemnicka, Karol Prałat, Artur Koper, Grzegorz Makomaski, Łukasz Majewski, Karolina Wójcicka, Katarzyna Ewa Buczkowska

**Affiliations:** 1 Faculty of Civil Engineering, Mechanics and Petrochemistry, Institute of Civil Engineering, Warsaw University of Technology, Łukasiewicza Street 17, 09-400 Płock, Poland; Justyna.Ciemnicka@pw.edu.pl (J.C.); Artur.Koper@pw.edu.pl (A.K.); Lukasz.Majewski@pw.edu.pl (Ł.M.); Karolina.Wojcicka@pw.edu.pl (K.W.); 2 Faculty of Civil Engineering, Institute of Chemistry, Mechanics and Petrochemistry, Warsaw University of Technology, Łukasiewicza Street 17, 09-400 Płock, Poland; Grzegorz.Makomaski@pw.edu.pl; 3 Department of Material Science, Faculty of Mechanical Engineering, Technical University od Liberec, Studentska 2, 461-17 Liberec, Czech Republic; katarzyna.ewa.buczkowska@tul.cz; 4 Department of Materials Technology and Production Systems, Faculty of Mechanical Engineering, Lodz University of Technology, Stefanowskiego 1/15, 90-001 Lodz, Poland

**Keywords:** ecological materials, strength, gypsum, microspheres, HEMC, aerogel, DSC

## Abstract

The paper presents an assessment of the impact of using additives on the strength of a binding material, i.e., building gypsum, and also the phase transformation that takes place in it. Microspheres, aerogel and polymer (HEMC) additives were added to a building gypsum slurry with a water to gypsum ratio of 0.75. In order to investigate their influence on bending strength, compressive strength, and the effect of high temperatures, differential scanning calorimetry (DSC), as well as tests of the multicomponent binder, were carried out in accordance with the applicable PN-EN 13279-2:2005 standard. The obtained test results allowed to determine that the used additives influenced the strength parameters of the obtained composites. It was shown that the applied additives decreased the compressive and bending strength of the modified gypsum. Despite these properties, the obtained gypsum materials are environmentally friendly because they reuse wastes, such as microspheres. Out of all the applied additives, the use of microspheres in an amount of 10% caused a decrease in the bending strength by only 10%, and an increase in the compressive strength by 4%.

## 1. Introduction

It is very important during the design and implementation of the entire investment process of a building to ensure proper conditions of the internal environment in terms of both health and comfort of use. Equally important is respect for the natural environment. Modern science and industry, which strive to minimize energy consumption in buildings, play an important role in reducing thermal losses in buildings.

The ecological construction industry involves the implementation of various methods of reducing energy consumption, which aim to reduce the use of conventional energy. These methods, therefore, focus on environmental protection, with this being achieved by:

− the use of energy from alternative (renewable) sources:
− the application of heat recovery systems;
− the introduction of unconventional methods of obtaining, storing and converting energy.

One way to save energy is to use appropriate insulation materials. Contemporary construction companies strive to produce building materials that have the best thermal parameters. Apart from materials that are typically used for insulation, i.e., mineral wool, polystyrene or polyurethane foam, the modification of basic construction materials should also be considered in order to obtain even better thermal properties for a building without losing their good strength properties.

There are many studies that have aimed to improve the thermal and strength properties of modified concretes [1,2,3,4]. Serina et al. investigated the effect of the addition of aerogel on the strength and thermal properties of concrete composites. The conducted tests of an aerogel-based mortar, which was prepared with the use of a recipe for a ultra-high-performance concrete (UHPC), showed that the increase in the volume of aerogel in mortar reduced the compressive strength of concrete mortar [1]. Gao at al., when conducting observations using a scanning electron microscope, showed that aerogel particles are stable during the hydration of cement materials, which suggests the possibility of combining aerogel and concrete materials in construction applications [2]. Kubissa et al. investigated the effect of adding microspheres to concrete. It was found that microspheres added to concrete reduce the compressive strength of all the modified concrete series presented in [3]. Jaskulski and Kubissa also showed that microspheres that are used as an additive to concretes have the potential to reduce thermal conductivity [4]. In the case of samples fully saturated with water, the obtained values of thermal conductivity, in almost all cases, did not differ from the values obtained for the reference samples. The lowest values of thermal conductivity (1.90 W/(m·K)) were obtained for the wet series with the addition of microspheres or a latex-based additive. In the case of the dried samples, the lowest thermal conductivity was obtained for samples with the addition of microspheres (1.37 W/(m·K)), and the highest for those with the addition of latex 1.78 W/(m·K)).

Although there are some studies concerning new cementitious materials, there are not many that deal with the properties of modified gypsum composites. The authors of other works [5,6] showed that the use of microspheres, aerogel and hydroxyethylmethyl cellulose (HEMC) in building gypsum significantly improves its thermal properties. By using additives, the values of thermal conductivity were reduced by up to 43% when compared to the reference gypsum samples without such additives [5,6]. However, the influence of the used additives on the mechanical properties of a multi-component gypsum binder was not demonstrated.

Heim et al. showed that the addition of hydroxyethyl methyl cellulose reduced the density of modified gypsum and changed its morphological structure [7]. The gypsum with HEMC content at a level of 1.0% resulted in a more than 20% lower thermal conductivity in comparison to the sample without HEMC. The obtained value of thermal conductivity was 0.2797 W/(m·K). At the same time, Mucha et al. discussed the influence of HEMC on the thermal parameters of modified gypsums. They showed that the use of this polymer in gypsum composites delayed the hydration process [8,9].

Aerogels are currently considered to be one of the most promising high-performance thermal insulation materials for building applications. Due to their thermal conductivity of up to 13 mW/(m·K), in the case of commercial products, they exhibit remarkable properties when compared to traditional thermal insulation materials [10]. A very interesting aspect of aerogels is that they can be produced as opaque, translucent or transparent materials, which allows for a wide range of possible building applications—especially when trying to use daylight and solar energy from solar radiation.

Another very interesting application of aerogels is their use in systems with phase change materials (PCMs). The authors of [11] investigated the thermal properties of a glass window, which consisted of glass, silica aerogel and a phase change material. Such a solution provided heat storage and its recovery, excellent thermal insulation, and daylight to an indoor environment. The results of the studies showed that the most important control parameters for such windows are the thermal conductivity and thickness of the silica aerogel layer. The authors experimentally determined the appropriate thickness of the aerogel layer that maximized the use of the latent heat of the PCM. For the tested climatic conditions, such a thickness was optimal in the range from 20 to 30 mm. It was found that the integration of the aerogel insulation, which has a low thermal conductivity coefficient, with the PCM-glass window system is an effective technology in cold regions of the world.

The authors of [12] designed and produced lightweight and heat-insulating foamed concrete with the addition of aerogel. The obtained product had a density of about 198 kg/m^3^, and a thermal conductivity of 0.049 W/(m·K). They emphasized that the resulting innovative composite can be used in eco-friendly and almost zero-energy buildings. The composite was successfully manufactured by adding super-insulating and nano-porous aerogels to microporous concrete.

Modified building materials, apart from their good thermal properties, should also have appropriate strength parameters with regards to bending and compressive forces, as well as have an adequate resistance to high temperatures.

Other works [5,6] showed that used additives had a very good effect on the improvement of the thermal properties of multi-component gypsum binders made with the use of wastes. Such composites can be seen to be environmentally friendly. The use of additives for the gypsum composites significantly reduced the thermal conductivity of the material. The greatest decrease in the value of λ was achieved with the use of 2% of the polymer in relation to the weight of the dry gypsum powder. The obtained conductivity value was 0.1948 W/(m·K). The addition of 15% of microspheres in relation to the gypsum caused a decrease in thermal conductivity by 25%, while the addition of 2% of aerogel in relation to the gypsum caused a decrease of this parameter by 16%.

The work in [13] presents results of thermal conductivity measurement of composites based on gypsum with addition of microencapsulated phase change material (PCM). Samples of different concentration of PCM and in the temperature range typical to building indoor conditions were analyzed. The investigation has shown strong influence of both PCM content and temperature on thermal conductivity. The thermal conductivity of 100% gypsum drops was from about 0.35 to about 0.25 W/(m·K) in the temperature range under consideration, i.e., from 19 °C to 30 °C. Similar trends are visible in the case of composites with PCM. It can be concluded that change of phase of PCM (melting) does not have a substantial influence on thermal conductivity. However, concentration of PCM has a significant impact on thermal conductivity. In the case of 30% concentration of PCM thermal conductivity for higher temperatures drops below 0.1 W/(m·K).

The aim of paper [14] consists on the improvement of thermal properties and lightness of gypsum plaster by combining it with granular cork collected from the Moroccan Maamora’s forest. This composite material is intended to be used in false ceiling such as cork–gypsum board instead of plasterboard; its use will be a contribution to improve energy efficiency in buildings. By varying the granular cork size, an experimental investigation of thermal proprieties of gypsum based composite material with embedded granular cork was mainly performed using the asymmetrical transient Hot Plate method.

They found thermal conductivity corresponding to high density of composites: 0.1255 W/(m·K) for 578 (kg/m^3^), 0.1869 W/(m·K) for 600 (kg/m^3^) and 0.1995 W/(m·K) for 864 (kg/m^3^).

In [15], the authors showed the effect of cellulose concentration on the gypsum-cellulose composites. The experiment clearly shows that, by increasing the cellulose fibers concentration, the density and the thermal conductivity of the gypsum-cellulose plates systematically decrease. The work in [16] proposes a hybrid numerical–experimental method for extracting thermal conductivity of various gypsum board products at elevated temperatures. Gypsum board is ubiquitously used in fire resistant construction, yet there is wide variability in reported values of thermal conductivity of gypsum at high temperatures. Given the effects of porosity, moisture and non-linear temperature distribution in gypsum under heating, direct measurement of thermal conductivity, e.g., by using the hot plate method, is not an easy task.

This paper presents the influence of microspheres, aerogel and polymer on the bending and compressive strength of modified gypsums, and also the influence of these additives on the resistance to high temperatures of modified gypsums. The aim of the study was to check how various additives used in gypsum composites will affect their mechanical properties. The comparison of the properties of gypsum materials with regards to the used additives (with diverse chemical and physical properties) has not been found in literature in one whole publication. The study also tried to find a correlation between the results obtained from the measurements conducted during the strength tests of the modified gypsums and the results obtained using differential scanning calorimetry (DSC). It can be assumed that the mechanical properties of modified building materials may correlate with the course of calorimetric curves. The authors of the study had previously conducted research concerning the influence of various additives on the thermal properties of modified gypsum. However, strength tests are necessary to fully understand the properties of innovative materials. Information on thermal and mechanical changes caused by gypsum additives consolidates the knowledge concerning these building materials. The aim of our extensive studies is to search for a correlation between the used additives and the performance properties of modern building materials.

## 2. Materials and Methods

### 2.1. Materials

#### 2.1.1. Building Gypsum

A commercially available building gypsum from Dolina Nidy (Pinczow, Poland) was used in the research. This powder meets the basic requirements for these types of building materials [17]. The basic properties of this material are summarized in Table 1.

#### 2.1.2. Microspheres

**Figure 1:**
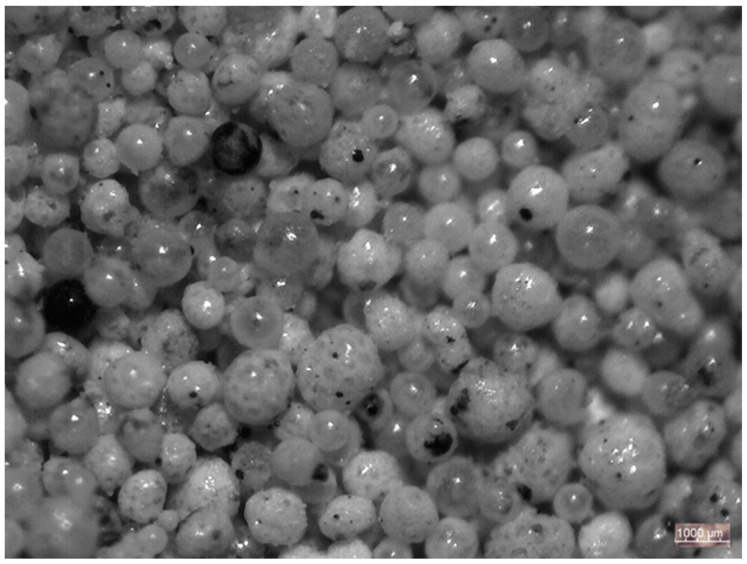
Microscopic image of the microspheres that were used in the tested composites.

Microspheres are particles that are a result of the conventional combustion of hard coal. The grains of microspheres have a wall thickness ranging from 2 to 30 µm [19]. Their walls are made of both amorphous and crystalline forms, while the inside of the grains is filled with carbon dioxide or nitrogen—gases generated in the process of coal combustion [20]. Microspheres can be used as additives to materials with organic binders. They are mainly used as a light filler for composites with PVC, epoxy resins, latex emulsions, rubber, or a polyurethane matrix [21,22,23,24,25,26,27,28]. Figure 1 shows microscopic images of typical microsphere grains.

Table 2 lists the selected physicochemical properties and chemical composition of the microspheres produced by Eko Export Inc. (Bielsko-Biala, Poland) that were used in the research.

#### 2.1.3. Aerogel

Aerogels are the lightest solid materials, and have the lowest thermal conductivity coefficient of 0.0089 W/(m·K) [29]. More than 99% of the mass of aerogel is air. The remaining part is silica. The fact that aerogels contain pores with dimensions from 2 to 50 nm means that they have very good acoustic properties. Extremely important features of aerogel are its non-flammability, non-toxicity, and thermal conductivity [30].

**Figure 2:**
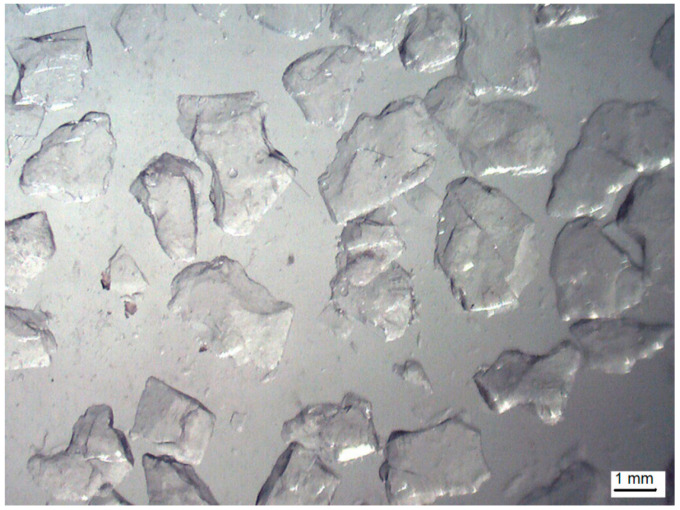
Microscopic image of the aerogel granulate used in the research [5].

For the purpose of the research, Lumira LA1000 aerogel granulate, manufactured by Cabot (Boston, MA, USA), was used as an additive to the gypsum composites. Figure 2 shows a microscopic image of the granulate, with its properties being summarized in Table 3.

#### 2.1.4. HEMC Polymer

**Figure 3:**
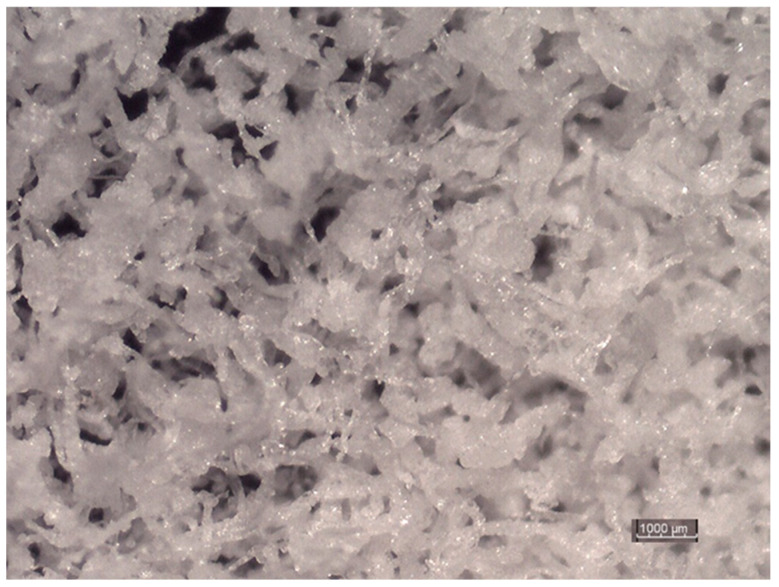
Microscopic image of the HEMC that was used in the experimental studies [5].

Hydroxyethyl methylcellulose is a polymer with thickening properties. Moreover, HEMC is used to disperse, bind, emulsify, and create films, and also retain water, in building mortars [32]. Due to its properties, it is widely used in water-based latex coatings, building materials, and printing inks, as well as oil drilling in the cosmetic industry [33,34,35,36,37]. In the pharmaceutical industry, hydroxyethyl methylcellulose is used as a hydrophilic gel matrix material, a pore-forming agent, and also a coating agent for sustained release formulations. Figure 3 shows the hydroxyethyl methylcellulose polymer powder that was used in the research (Sigma Aldrich, Warsaw, Poland).

### 2.2. Methods

For the purpose of the research, the gypsum slurry (with a water to gypsum ratio of 0.75) was enriched with additives in the following mass amounts in relation to the dry gypsum powder: microspheres (5%, 10%, 15%), aerogel and polymer (0.5%, 1%, 2%). The amounts of used additives were related to the high cost of the aerogel and polymer, which is a significant aspect in industrial conditions. The increased amount of the addition of microspheres in relation to other additives was caused by the desire to incorporate as much of this waste as possible, which in turn has a positive impact on the natural environment. Moreover, a higher amount of aerogel additive caused problems with the mixing process. The obtained composites were tested with regards to their bending and compressive strength. The differential scanning calorimeter (DSC) test was also carried out.

#### 2.2.1. Bending and Compressive Strength Tests

The composites prepared in accordance with the developed recipe were placed in 40 mm × 40 mm × 160 mm molds and left for 24 h until they were fully set. After demoulding, the formed bars were conditioned at a temperature of 20–22 °C and in 52 ± 2% humidity for 28 days. After this time, the test specimens were additionally dried at 65 °C for 7 days in order to remove as much water as possible.

**Figure 4:**
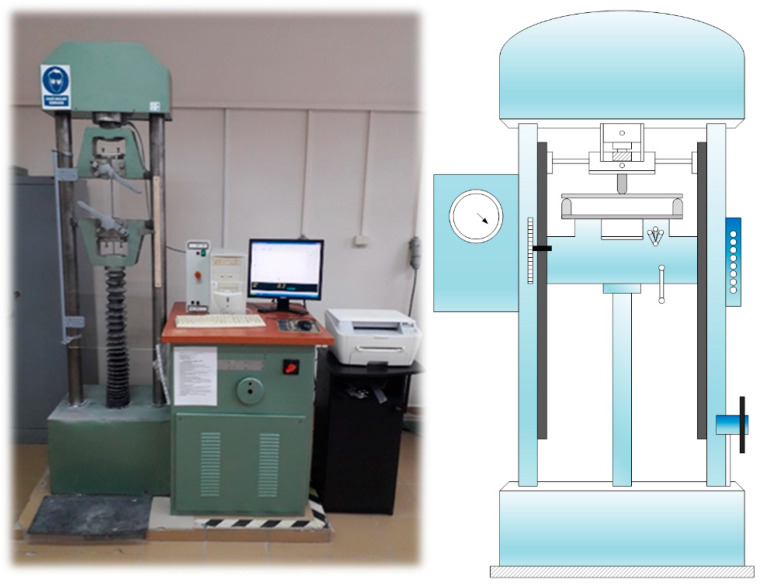
Heckert ZD10/90 measuring device for the strength tests of building materials.

The dried samples were tested with regards to their bending strength using a Heckert ZD10/90 testing device (WMW Fritz Heckert, Leipzig, Germany), in which the force is applied linearly. The test was carried out in accordance with the PN-EN 13279-2: 2005 standard. The speed of retraction of the lower mounting head was set at 1 mm/min. The beams’ halves, which were formed after the bending strength tests, were tested with regards to their compressive strength in accordance with the provisions of the standard. Figure 4 shows the stand for conducting the strength tests of the modified gypsum specimens.

#### 2.2.2. Differential Scanning Calorimetry

**Figure 5:**
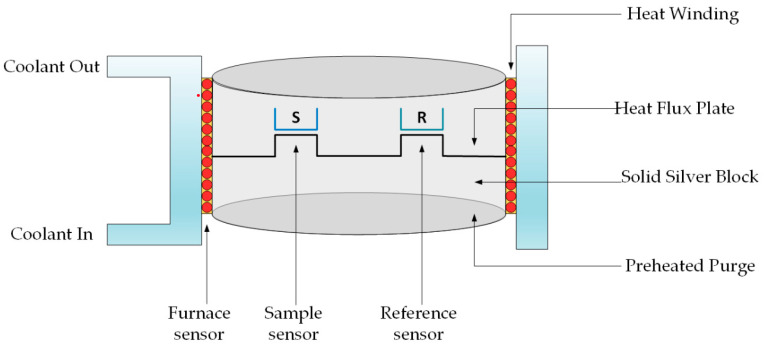
The scheme of operation of the differential scanning calorimeter (DSC).

Differential scanning calorimetry (DSC) enables the thermal effects accompanying the processes that occur during heating or cooling a specimen of a given substance to be tested. The DSC test is a direct measurement of the heat generated by chemical reactions and physical processes. The determination of the heat of transformation allows the values of other thermodynamic quantities to be determined [38]. During tests in a differential scanning calorimeter (Figure 5), containers with the tested samples and the reference sample are analyzed under the same conditions in accordance with the established temperature program—measuring the temperature difference between them. The reference specimen is usually an empty container [39]. As a result of the conducted measurements, a graph of the dependence of the supplied energy as a function of temperature is obtained. The amount of heat supplied to equalize the temperatures of the reference sample and the tested material is registered as a peak, and the area under it is equal to the enthalpy of the transformation [38,39,40,41,42].

## 3. Research Results and Analysis

**Figure 6:**
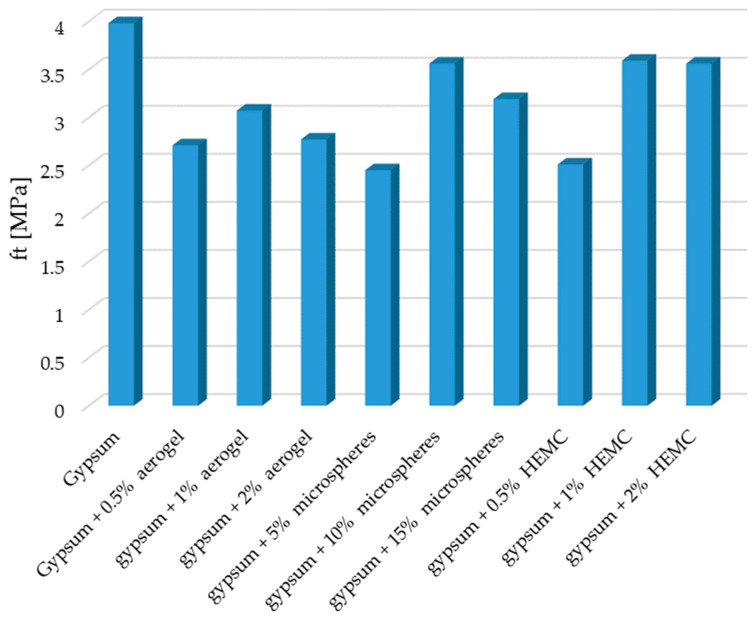
Bending strength ft of the modified gypsum composites.

The use of additives reduced the bending strength of the composites. The composite with the addition of 5% (by mass) of microspheres was characterized by the lowest bending strength. There was a decrease in strength by 38.4% when compared to the reference sample. The smallest influence on the bending strength had the addition of 1% (by mass) of HEMC and the addition of 10% (by mass) of microspheres. The decrease in the bending strength for this composite was by about 10% in both cases when compared to the gypsum sample without the additives. A slightly greater decrease in strength was noted in the case of the samples containing the addition of HEMC in the amount of 2% by weight. In the case of the remaining samples, the decrease in strength was more significant and ranged from 11% to 37%. The obtained results of the examination of the building gypsum composites modified with additives with regards to bending force are presented in Figure 6.

**Figure 7:**
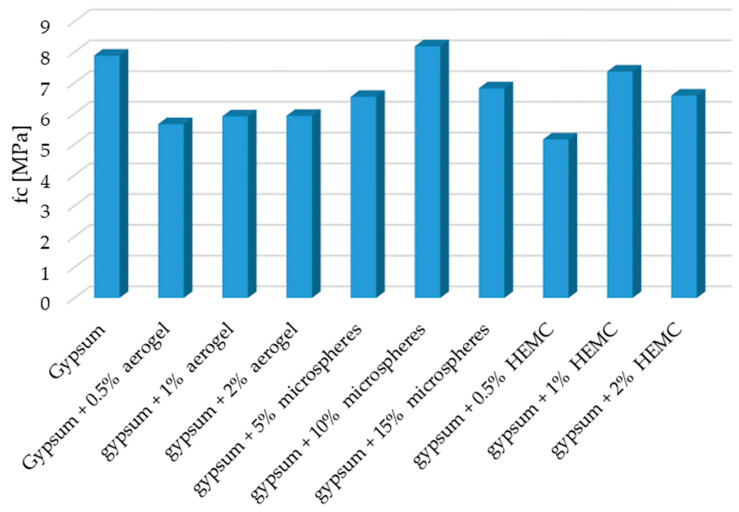
Compressive strength fc of the modified gypsum composites.

After the bending strength test, all the specimens were tested in terms of their compressive strength. The obtained results are shown in Figure 7.

As was the case with bending strength, a reduced compressive strength of the composites was noted. The composite with the addition of 0.5% by mass of HEMC was characterized by the lowest resistance to compression. Its strength was lower by 34.6% when compared to the reference sample.

**Figure 8:**
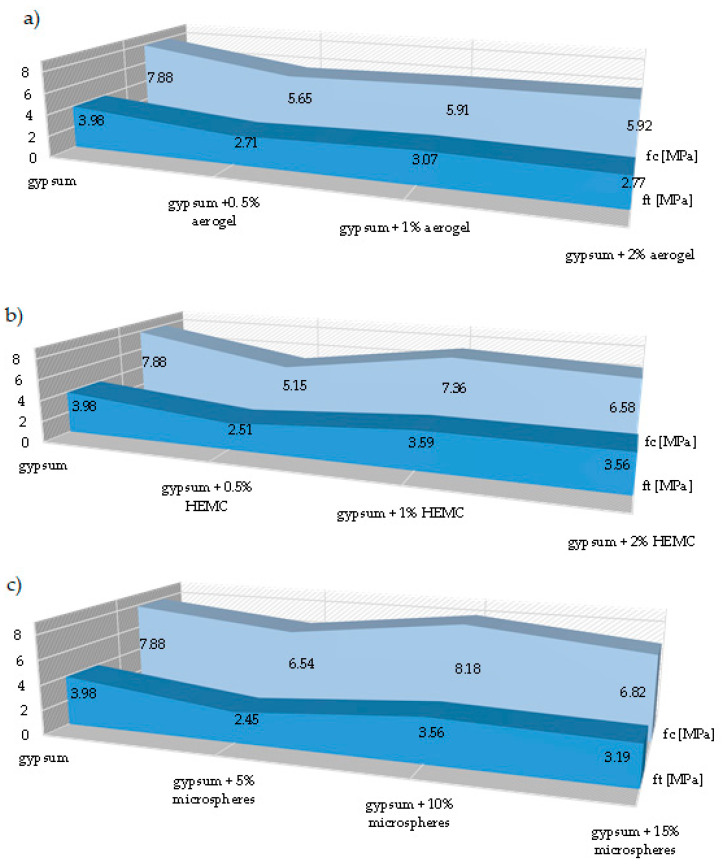
Bending strength ft and compressive strength fc of the gypsum with regards to the amount of used additive: (**a**) aerogel, (**b**) polymer, and (**c**) microspheres.

The addition of 1% by weight of HEMC had the least negative effect on the bending strength. The decrease in the resistance to bending for this composite was about 6.6% when compared to the gypsum sample without the additives. The addition of 10% by mass of microspheres increased the compressive strength of the composite by 4% when compared to the reference sample, which is an interesting exception in the present study (Figure 8a–c). The obtained test results show that the addition of additives to gypsum reduces its bending and compressive strength when compared to pure gypsum. However, the authors point out that in the case of aerogel and polymer, the composites with the lowest amount of them had the lowest bending and compressive strength, while the composites with 1% of aerogel and 1% of HEMC exhibited the highest strength.

**Figure 9:**
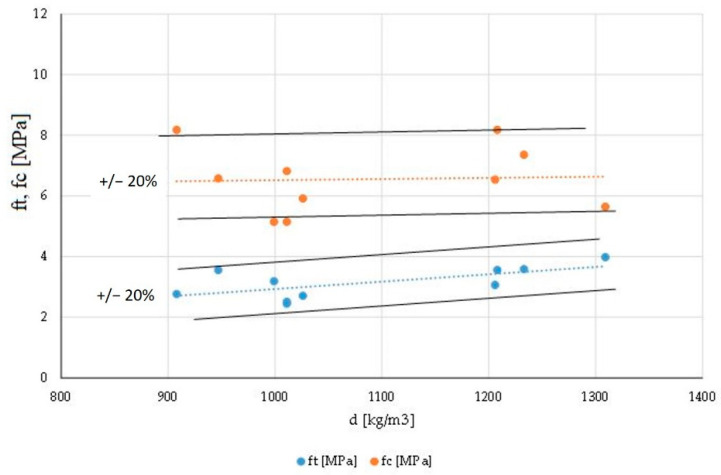
The dependence between the bending and compressive strength of the tested materials as a function of density.

The reduction in the strength of the composites against bending and compressive forces is related to the changing morphological structure of the sample. Strength is significantly influenced by changes in density and porosity. As a result of the applied additives, a decrease in the density of the obtained material was noticed in all the cases. Figure 9 shows the graphs of the dependence of the bending strength and compressive strength as a function of the density of all the modified gypsums. All the experimental points do not differ by more than ±20% from the designated trend lines of (1) and (2).

*f_c_* = 0.0004 × *d* + 6.1408 (1)

*f_t_* = 0.0024 × *d* + 0.4924 (2)

Mucha et al. [9] came to similar conclusions as the authors of this publication, whilst also stating that an increased porosity of the material resulted in an increase in its brittleness. Many works on the influence of additives on the microstructure and strength of gypsum were conducted in the second half of the twentieth century. The negative impact of the use of additives in gypsum composites was found, among others, by Yamada et al. [43], as well as Murat and Jeandot [44]. Current research focuses on examining the possibility of using various types of fibers to increase the mechanical strength of gypsum. Serna Jara et al. [45] proved in their research that the addition of graphene to gypsum slurry increased its resistance to mechanical forces by almost 13% when compared to the reference sample. A similar effect of strengthening a building material was noted for cement mortars modified with basalt fibers [46].

The authors of this study noticed that the composites with 1% of aerogel and polymer, and the composites with 10% of microspheres, were characterized with the highest density in each series. There is an assumption that this is related to the binding of water in the composite, which was indicated by the results of the research carried out with the use of differential scanning calorimetry (DSC). On the basis of the collected information, it can be assumed that there is a certain optimal concentration of the additive, the exceeding of which causes the deterioration of the composite’s strength. In order to confirm and complete this thesis, research on the application of additives to gypsum with regards to other percentages of them in samples should be continued in the future.

The examination of gypsum using the differential scanning calorimetry method allows valuable information on the behavior of the material under the influence of specific temperatures to be obtained. In the literature, there are many results of DSC studies for various types of gypsums and their composites [47,48,49].

Based on the course of the DSC curves for the gypsum, gypsum with aerogel, gypsum with polymer, and gypsum with microspheres, it was found that transitions that are characteristic for gypsum occurred as an effect of temperature. Three characteristic stages of dehydration can be noticed:

− endothermic transformation related to dehydration according to Equation (3):
  CaSO_4_·2H_2_O → β-CaSO_4_·1/2 H_2_O + 3/2H_2_O ↑ (3)
− endothermic transformation related to dehydration according to Equation (4):
  β-CaSO_4_·1/2 H_2_O → β-CaSO_4_-III + 1/2H_2_O ↑ (4)
− exothermic transformation related to phase transformation (5):
  β-CaSO_4_-III → β-CaSO_4_-II (5)

**Figure 10:**
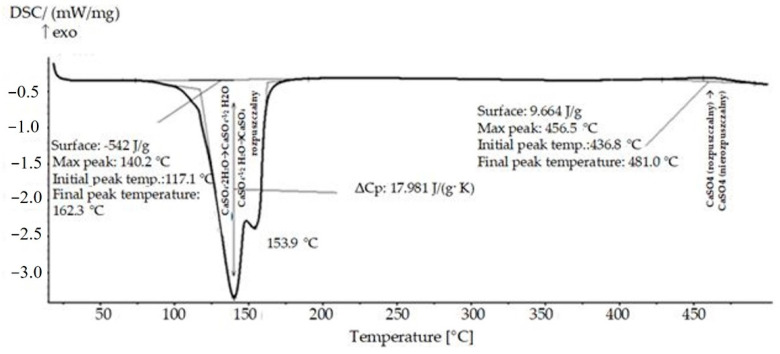
DSC curve of the pure gypsum.

Figure 10 shows the DSC curve of the pure gypsum. The endothermic transformation associated with the dehydration process was found to begin at 117.1 °C (Equation (3)), and continued at 153.9 °C (Equation (4)). Chandara et al. [50] obtained similar temperature values for the dehydration of gypsum. The beginning of the process was observed at a temperature close to 110 °C. The beginning of the second peak (related to further dehydration of the samples) was noticed at the temperature of 160 °C [50]. Doleželová et al. [51] showed that the dehydration process of gypsum takes place at a temperature below 200 °C. The differences in the calorimetric tests may result from the different purity of the gypsum used in the tests and the different degree of hydration of the samples. The effects occurring in Equations (3) and (4) are most often accompanied by a weight loss of approximately 20%.

**Figure 11:**
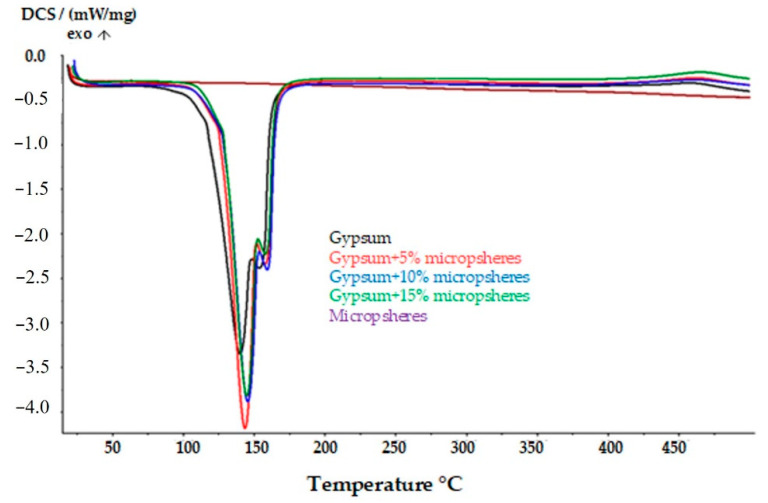
Comparison between the DSC curves of the gypsum composites with the addition of different amounts of microspheres and the DSC curve of the pure gypsum.

Based on the DSC investigation (Figure 11), it was found that the heat of transformation of the pure gypsum was equal to 542 J/g. The course of the curve shows that the specific heat of the sample containing the pure gypsum is equal to 17,981 J/(g·K). The exothermic transformation of the sample started at 436.8 °C. Table 4 summarizes the parameters of the phase transformations of all the modified samples.

Figure 11 shows a comparison between the DSC curves of the composites with different amounts of microspheres and the DSC curve of the pure gypsum. An increase in the peak area of complex II was observed in the modified materials, which gives the basis for the conclusion that the presence of microspheres increases the water binding in the material. The phase transformation of the gypsum modified with the additives occurs at higher temperatures. By analyzing the DSC curves of the composites modified with the additives, it was noticed that this transformation takes place at temperatures close to 436 °C. The phase transition temperature in the composites with the other additives was about 380 °C. Therefore, it can be assumed that the presence of microspheres improves the gypsum’s resistance to high temperatures.

**Figure 12:**
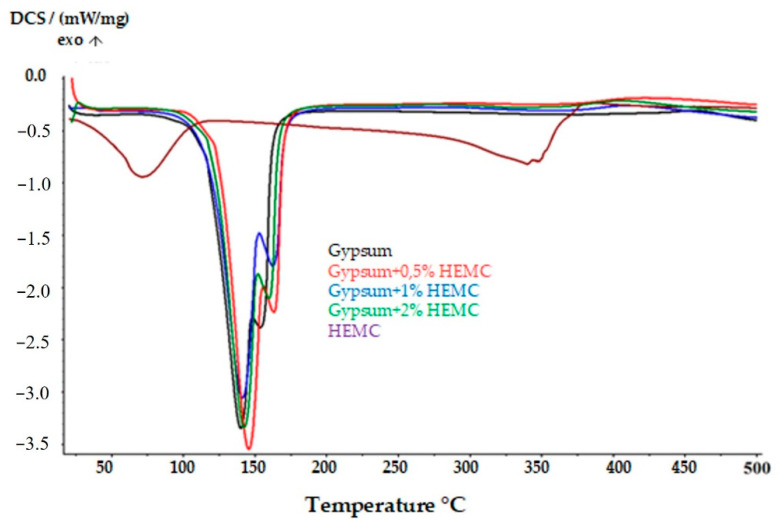
Comparison of the DSC curves of the gypsum composites with the addition of various amounts of polymer and the DSC curve of the pure gypsum.

A similar study was carried out on the gypsum composites containing the polymer additive. Figure 12 shows a comparison between the DSC curves of the gypsum, polymer and gypsum composites with the addition of HEMC. It can be seen that the transformation related to the dehydration of the gypsum–polymer composites occurs in a similar temperature range as the gypsum itself (117–128 °C). A difference, however, can be seen in the temperatures associated with the phase transformation. It was observed that the addition of polymer to the gypsum reduces the temperatures (by almost 50 °C) at which the exothermic transformation occurs. Therefore, it should be assumed that the addition of polymer to gypsum lowers its resistance to high temperatures. Moreover, it was found that the admixture of the polymer causes more water to be retained in the material when compared to pure gypsum.

**Figure 13:**
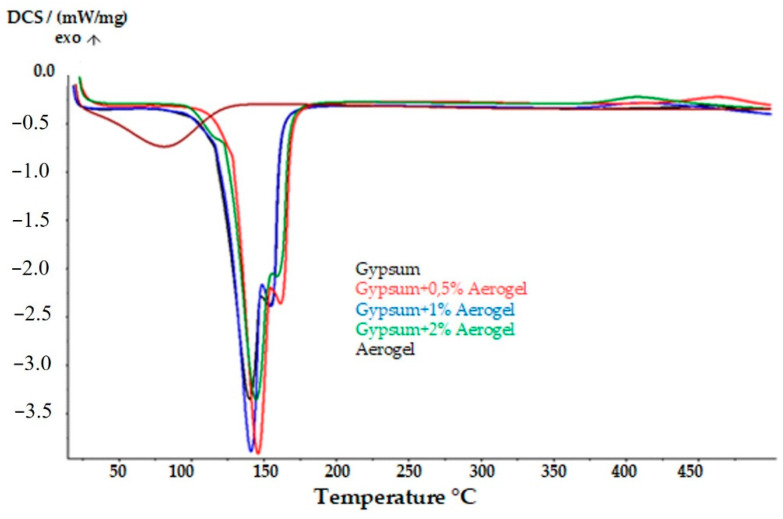
Comparison of the DSC curves of the gypsum composites with the addition of various amounts of aerogel and the DSC curve of the pure gypsum.

Figure 13 presents the DSC curves of the gypsum, as well as its mixtures with aerogel. It was found that the addition of the aerogel lowers the temperatures in which the phase transformation takes place by nearly 50 °C. Therefore, it can be assumed that this reduces the material’s resistance to high temperatures. Moreover, based on the observed increase in the endothermic peak area, there is a high probability that the addition of the aerogel causes water retention in the composite material, and in turn a decrease in its strength.

The positive effect on the strength of building materials with the addition of microspheres has also been presented in other scientific papers. Shahidan et al. [52] investigated the properties of lightweight foam concrete, in which Portland cement (OPC) was replaced with hollow glass microspheres (HGMs) in an amount of 0%, 3%, 6%, and 9%. These studies showed that the number of hollow glass microspheres plays an important role when determining the strength, water absorption, and thermal insulation of foam concrete. The optimal amount of added HGMs turned out to be 3%, which increased the compressive strength from 1.8 MPa to 2.2 MPa. The use of 9% of microspheres caused a deterioration of the compressive strength when compared to the reference sample by 0.1 MPa.

Inozemtcev et al. [53] presented the results of research concerning the physico-mechanical and operational properties of high-strength lightweight concrete, and also the influence of the nano-modifier on these properties. A nano-modifier and a method of its application were proposed to improve the properties of lightweight concrete with hollow microspheres by 10–25%. The developed composite has a dense and strong structure that is resistant to intense cracking. The use of a nano-modifier enabled the modulus of elasticity to be increased by 13–36% (equal to 6.2–8.5 GPa, depending on the average density). Chen J. [54] proved in his research that the addition of fly ash microspheres and condensed silica dust can significantly increase bulk density, and therefore also increase fluidity and strength. The addition of microspheres in the amount of 20%, and condensed silica dust in the amount of 10% increased the strength of the composite from 90 MPa to 159 MPa.

The authors of this study showed that various additives reduce the strength of gypsum composites. The conducted tests showed that the use of 10% of the amount of microspheres had a positive effect on the compressive strength of the obtained composite (increased the strength to 8.18 MPa) when compared to the reference sample, in which the strength was equal to 7.88 MPa. The bending strength values were 3.59 MPa for the modified sample, and 3.98 MPa for the reference sample. The improvement of the thermal and strength properties of building materials with the use of an additive in the form of microspheres requires further research in order to determine the effect of this additive on the properties of other building materials, including gypsum.

On the basis of the presented information from the DSC tests, it can be assumed that there is a certain optimal amount of additive in gypsum, the exceeding of which causes the deterioration of the composite’s strength. Differential scanning calorimetry tests confirmed the observations made during the strength tests, in which the smallest decrease in the bending and compressive strength was noticed in the case of using 1% of HEMC and 1% of aerogel. The DSC tests completed and confirmed the assumptions resulting from the mechanical measurements. In the future, the authors plan to continue their research concerning the use of additives in modified gypsum materials using different percentages in the samples.

Based on the authors’ previous research [5,6,55] and that of different authors [13,14,15,16], it was noticed that the used additives significantly improve the thermal insulation properties of gypsums. Current research shows that these additives deteriorate their mechanical properties. Therefore, a compromise should be found for improving the thermal properties of gypsum composites without significantly reducing their strength properties.

## 4. Conclusions

- The obtained test results allow for the conclusion that the addition of different additives to gypsum causes a change in the gypsum’s resistance to compressive and bending forces. The most unfavorable effect on the bending strength of gypsum was the addition of 5% of microspheres, which caused a decrease in strength by 38.4%.
- The addition of 10% by mass of microspheres increased the compressive strength of the composite by 4%.
- A great advantage of using composites is the possibility of managing waste such as microspheres, which, when used in an optimal amount, improve the thermal properties and the compressive strength of the composites. The management of waste from combustion processes in power plants is of great pro-environmental importance.
- The use of differential scanning calorimetry in the studies of the gypsum containing different additives enabled the temperature and heat of phase transformations, as well as the specific heat of materials, to be determined. The effect of adding aerogel, microspheres or polymer into gypsum on the content of physically bound water, and thus on the value of transformation heat and thermal conductivity, was demonstrated.
- The use of the additives in the building gypsum caused water retention in the composite, which was observed as an increase in the surface area of the peak that is associated with the gypsum’s dehydration. The obtained DSC results also allow for the statement that there is an increase in resistance to high temperatures of composites modified with different additives.
- The DSC tests confirmed and supplemented the information that was obtained during the strength tests.
- The applied additives are very good materials that improve the thermal properties of the obtained gypsum composites. However, they reduce the bending and compressive strength of these composites.

## Figures and Tables

**Table 1:** Physical properties of the building gypsum applied in the research [17,18].

Building Material	Relative Density *d* [kg·m^−3^]	Bulk Density *d_B_* [kg·m^−3^]	pH	Color	Appearance	Setting Time [min]
Building gypsum	2300	900	7–8	grey	grey–yellow powder	3

**Table 2:** Composition and selected physical properties of the microspheres that were used to produce the gypsum composites.

Properties of Microspheres	
Content of Al_2_O_3_	34–38%
Content of Fe_2_O_3_	1–3%
Content of SiO_2_	50–60%
Content of K_2_O	0.1–2%
Content of CaO	1–4%
Content of MgO	0.2–2%
Content of TiO_2_	0.5–3%
Melting temperature	Above 1600 °C
Total mass	0.378 g/cm^3^
Grain diameter	150–300 μm

**Table 3:** Properties of the aerogel granules used in the research [31].

Properties of Lumira LA1000 Aerogel	
Grain size range	0.7–4 mm
Pore diameter	ok. 20 nm
Grain density	120–150 km/m^3^
Bulk density	65–85 kg/m^3^
Surface area	600–800 m^2^/g
Thermal conductivity	18–23 mW/(m·K)

**Table 4:** Parameters of the phase transformation of the tested composites read from the DSC curves.

	Temperature at the Beginning of the Dehydration Process [°C]	Heat of Transformation [J/g]	Specific Heat [J/(g·K)]	Temperature at the Beginning of the Exothermic Transformation [°C]
Gypsum	117.1	542	17.981	436.8
Gypsum +5% microspheres	126.6	546.1	23.056	436.8
Gypsum +10% microspheres	128.6	517.0	21.184	429.1
Gypsum +15% microspheres	128.1	523.3	21.032	441.8
Gypsum +0.5% HEMC	125.1	568.8	19.439	383.2
Gypsum + 1% HEMC	118.1	533.3	16.358	382.2
Gypsum +2% HEMC	121.5	539.5	18.268	370.8
Gypsum +0.5% aerogel	128.4	551.6	21.48	441.4
Gypsum +1% aerogel	121.3	554.2	21.318	383
Gypsum +2% aerogel	125.5	565.3	18.311	382.2

## Data Availability

The data presented in this study are available on request from the corresponding author.
